# Radioimmunotherapy of prostate cancer targeting human kallikrein-related peptidase 2

**DOI:** 10.1186/s13550-016-0181-z

**Published:** 2016-03-17

**Authors:** O. Vilhelmsson Timmermand, E. Larsson, D. Ulmert, T. A. Tran, SE. Strand

**Affiliations:** Department of Clinical Sciences Lund, Oncology and Pathology, Lund University, Barngatan 2:1, Lund, S-21185 Sweden; Department of Clinical Sciences Lund, Medical Radiation Physics, Lund University, Barngatan 2:1, Lund, S-21185 Sweden; Department of Surgery (Urology), Memorial Sloan-Kettering Cancer Center, 1275 York Avenue, New York, NY 10065 USA; Lund University Bioimaging Center, Lund University, Klinikgatan 32, BMC D11, Lund, S-22242 Sweden

**Keywords:** Prostate cancer, Radioimmunotherapy, Human kallikrein-related peptidase 2, hK2, 11B6, ^177^Lu, ^177^Lu-m11B6, Dosimetry

## Abstract

**Background:**

Prostate cancer ranks as the second most lethal malignancy in the Western world. Previous targeting of prostate-specific antigen and human kallikrein-related peptidase 2, two related enzymes abundantly expressed in prostatic malignancies, with radioimmunoconjugates intended for diagnostic purposes, have proven successful in rodent prostate cancer (PCa) models. In this study, we investigated the uptake and therapeutic efficacy of ^177^Lu-m11B6, a human kallikrein-related peptidase 2 (hK2)-targeting radioimmunoconjugate in a pre-clinical setting.

**Methods:**

The murine 11B6 antibody, m11B6, with high affinity for hK2, was labeled with ^177^Lu. Therapy planning was done from a biokinetic study in LNCaP xenografts, and therapeutic activities of ^177^Lu-m11B6 were administered to groups of mice. Body weight and general conditions of the mice were followed over a period of 120 days.

**Results:**

The tumor uptake in LNCaP xenografts was 30 ± 8.2 % injected activity per gram 1 week post-injection. In vivo targeting was hK2-specific as verified by a 2.5-fold decrease in tumor uptake in pre-dosed xenografts or by a fourfold lower tumor accumulation in hK2-negative DU 145 xenografts. Therapy showed a dose-dependent efficacy in LNCaP xenografts treated with ^177^Lu-m11B6. No therapeutic effect was seen in the control groups. The median survival for the lowest given activity of ^177^Lu-m11B6 was 88 days compared to that of 38 days in mice given labeled non-specific IgG. For the higher administrated activities, total tumor regression was seen with minimal normal organ toxicity.

**Conclusions:**

We have proven the possibility of radioimmunotherapy targeting hK2 in subcutaneous prostate cancer xenografts. ^177^Lu-m11B6 exhibited high therapeutic efficacy, with low observed toxicity. Additionally, an evaluation of the concept of pre-therapy planning using a dosimetry model was included in this radioimmunotherapy study.

## Background

Prostate cancer (PCa) is the most commonly diagnosed cancer in men, and the second most lethal cancer among North American men [1]. Localized PCa is treated with surgery and/or radiation therapy. For those patients who have advanced androgen sensitive disease, the standard treatment is usually different forms of androgen deprivation therapy [2]. If the disease progresses to castration-resistant prostate cancer (CRPC), a state of resistance to the therapeutic regiment of serum androgen suppression, the prognosis becomes poor, with an expected survival for patients with metastases of less than 19 months [3]. Resistance to androgen suppression is complex and relies on, e.g., changes in androgen receptor (AR) sensitivity, AR specificity, AR ligand independence, or bypassing of the AR pathway [4]. Commonly, CRPC is treated with the continuation of androgen deprivation, chemotherapy, and external beam radiation therapy (EBRT). However, there are also several new approaches with promising results for combating CRPC, ranging from anti-androgen synthesis therapy to immunotherapy [5]. Radionuclide therapy with the α-emitting bone seeker ^223^Ra (Xofigo, Bayer) has shown encouraging results with an increase of median overall survival of 3.6 months [6]. Also, prostate-specific membrane antigen (PSMA)-targeted radioimmunotherapy (RIT) has been extensively studied. ^177^Lu-labeled J591 targeting the external domain of PSMA has shown efficacy in phase II clinical trials [7], which is encouraging for the development of novel RIT approaches in CRPC treatments. However, today, the development of resistance to the therapeutic regimens eventually renders all therapies suboptimal.

RIT is one of few therapeutic options, where the outcome and tolerable dose can be better predicted for each individual treatment based on dosimetry [8]. This is possible since the absorbed dose to both normal organs and tumor can be correlated to the observed biokinetics. Usually, medium energy β^−^-emitters are the choice of therapeutic radionuclides. The resulting crossfire effect circumvents any need for targeting all malignant cells and reduces the impact of low tumor penetration and heterogeneous antigen localization and therefore gives better tumor response [9]. Even though RIT of solid cancers is not common and could still be considered to be in evaluation [10], a RIT rationale against PCa could work since PCa is a relatively radiosensitive disease and since metastatic PCa localizes to tissue that receive high levels of circulating antibodies, such as bone marrow and lymph node metastases [11].

Human kallikrein-related peptidase 2 (hK2) is a prostate-specific serine protease normally highly specific to the prostate [12] with low or no expression in other organs. hK2 shares about 80 % homology with prostate-specific antigen (PSA) and is encoded by the human kallikrein 2 gene (KLK2) [13, 14]. KLK2 is an AR-regulated gene, and since AR signaling is in general retained in metastasized PCa and CRPC [15, 16], targeting a protein downstream of the AR, like hK2, could be beneficial. Data from patient tissues has suggested that the relative expression of KLK2 is increased in malignant tissue compared to benign and healthy prostatic tissue, and the intensity of hK2 immunostainings in, e.g., lymph node metastasis correlates better with PCa tumor grade than that of PSA [13, 14].

Our group recently published a work on a new radioimmunoconjugate based on the ^111^In-labeled murine monoclonal antibody 11B6 (m11B6) suitable for molecular imaging of free, not associated with protease inhibitors, hK2 in PCa [17]. 11B6 is an IgG_1_ with a high affinity and specificity for free hK2 [18]. ^111^In-labeled m11B6 showed high tumor accumulation in both subcutaneous (s.c.) as well as bone-enclosed PCa xenografts, mimicking metastasized PCa [17]. This could translate to high absorbed doses to the tumor using a β-emitter like ^177^Lu. Additionally, ^177^Lu has a gamma component which allows the in vivo distribution of this radionuclide to be followed using single photon emission computed tomography (SPECT).

Here, we present results on the therapeutic efficacy and the biokinetics of m11B6 labeled with ^177^Lu in a pre-clinical setting. This is, to our knowledge, the first data on RIT targeting a secreted antigen belonging to the kallikrein-related peptidase family. Additionally, we evaluated therapy planning with a dedicated dosimetry model in an attempt to define the pre-clinical therapeutic window of ^177^Lu-labeled m11B6.

## Methods

### Conjugation and radiolabeling

#### Conjugation

Conjugation was performed as previously described [17]. Shortly, m11B6 (provided by the University of Turku, Finland) in 0.07 M sodium borate buffer (Sigma Aldrich, St Louis, MO, USA), pH 9.2, was concentrated on an Amicon Ultra-2 centrifugal filter, 2 mL, 100 K, (Millipore, Billerica, MA, USA) and conjugated at 40 °C with the chelator CHX-A''-DTPA (Macrocyclics, Dallas, TX, USA) in a chelator to antibody molar ratio of 3:1 for 4 h. The reaction was terminated and CHX-A''-DTPA-m11B6 was separated from free chelate by size-exclusion chromatography using a NAP-5 column (GE Healthcare, Uppsala, Sweden), equilibrated with 20 mL of 0.2 M ammonium acetate buffer pH 5.5. The conjugated antibody was kept at −20 °C for labeling.

#### Radiolabeling

Approximately 600 μg CHX-A''-DTPA-m11B6 in ammonium acetate buffer pH 5.5 (~200 μL) was mixed with 200–800 MBq ^177^LuCl_3_ (IDB Holland, Petten, Holland), ~5–50 μL depending on the specific activity, the final volume was set to 0.5 mL by adding ammonium acetate buffer (pH 5.5). The final solution was incubated at room temperature for 2 h. The labeling was terminated by purification on a NAP-5 column (Ge Healthcare) equilibrated with PBS (Sigma Aldrich). Labeling efficiency and labeling kinetics were monitored with ITLC strips (Biodex, Shirley, NY, USA), eluted with 0.2 M citric acid (Sigma Aldrich) and evaluated with a PhosphorImager system (Perkin Elmer, Waltham, MA, USA) using OptiQuant as analysis software (Perkin Elmer). The labeled immunoconjugate is from now on denoted ^177^Lu-m11B6. Additionally, for therapy with non-specific mouse IgG, 18765 (Sigma Aldrich) was conjugated and radiolabeled in the same way as m11B6.

### In vitro stability studies

The stability of the labeled conjugate was tested in triplicates of 10 μL by incubating in 100 μL PBS or EDTA solution with a final 500:1 M ratio of EDTA to chelate (Sigma Aldrich) at 4 °C up to 2 weeks. Also, the stability was tested in 100 μL of mouse serum and in PBS as a control, at 37 °C. The serum samples were taken at 1 week, and the EDTA samples up to 2 weeks after incubation. The EDTA samples were analyzed using ITLC strips and serum samples were mixed with NuPAGE® LDS Sample Buffer (Thermo Fisher Scientific, Waltham, MA, USA) and deionized water and heated at 70 °C according to the manufacturer’s instructions for separation on a NuPAGE®Bis-Tris Gel (Thermo Fisher Scientific). The radioactivity distribution was analyzed on a PhosporImager system as above.

### Cell lines

LNCaP and DU 145 were purchased from American Type Culture Collection (ATCC, Manassas, VA, USA). LNCaP cells express hK2. DU 145 cells were used for controls in this study, since they produce very small amounts of endogenous hK2 [19]. For simplicity, these cell lines will be described as hK2 positive (LNCaP) and hK2 negative (DU 145). Cells were cultured in RPMI 1640 medium (Thermo Fisher Scientific) supplemented with 10 % fetal bovine serum (Thermo Fisher Scientific) with 100 IU/mL penicillin and 100 μg/mL streptomycin (Thermo Fisher Scientific). The cells were maintained at 37 °C in a humidified incubator at 5 % CO_2_ and were detached with trypsin-EDTA solution (Thermo Fisher Scientific).

### Animal studies

All animal experiments were performed with the approval of the local Ethics Committee for Animal Research (Malmö-Lund University, Sweden). All applicable international, national, and institutional guidelines for the care and use of animals were followed. Male immunodeficient nude mice aged 6–8 weeks old (NMRI-Foxn1^nu^/Foxn1^nu^), purchased from Charles River (Charles River, Wilmington, MA, USA), were used. The mice were inoculated in the right flank by a subcutaneous injection of 5–8 × 10^6^ cells in a 200 μL cell suspension of 1:1 mixture of medium and Matrigel (BD Biosciences, San Jose, CA, USA). Tumors were allowed to develop for 6–8 weeks.

#### Small-animal SPECT imaging

The mice carrying LNCaP xenografts (*n* = 9) or DU 145 xenografts (*n* = 4) were injected intravenously (i.v.), through tail-vein injections, with ^177^Lu-m11B6 (7.9 ± 0.69 MBq, 30 μg mAb, in approximately 100 μL PBS) for small-animal SPECT/CT (Bioscan) imaging, using the NSP-106 multi-pinhole mouse collimator. Energy windows of 20 % were centered over the 113 and 208 keV energy peaks [20]. SPECT data were reconstructed using HiSPECT software (SciVis, Goettingen, Germany). Three mice belonging to the LNCaP xenograft group had been pre-dosed 24 h prior to injection with 1 mg of unlabeled m11B6. These animals were imaged at 72 h or 168 h p.i. and euthanized and dissected as follows: 72 h (LNCaP, *n* = 3; pre-dosed, *n* = 3; and DU 145: *n* = 4) or 168 h (LNCaP, *n* = 3). The organs and tissues were measured for activity content in an automated well counter with a 3-in. NaI(Tl) detector (1480 WIZARD, Perkin Elmer), as described below. SPECT data analysis and quantification were done in InVivoScope 2.0 software (inviCRO, Boston, MA, USA), and regions of interest (ROIs) were drawn using the CT image as an anatomical reference.

#### Biodistribution studies

The in vivo hK2 specificity of ^177^Lu-m11B6 was investigated by pre-dosing with cold m11B6 in LNCaP xenografts 24 h prior to injection with 1 mg of unlabeled m11B6 or by administrating labeled m11B6, in hK2-negative DU 145 xenografts. The activities given were 7.9 ± 0.69 MBq (specific activity, 0.26 MBq/μg). The blood and organs (including tumor) were collected and weighed, and the number of counts was measured in a NaI(Tl) well counter (1480 Wizard, Wallac, Perkin Elmer). The number of counts was corrected for decay and, using a volume-dependent sensitivity factor, converted to activity. The organ uptake values were calculated as percent injected activity per gram tissue (%IA/g).

### Dosimetry and therapy planning

#### Dosimetry

The therapy dose planning was based on the biokinetic data obtained with ^111^In-m11B6 in a previously published study [18] and biokinetic data acquired following the administration of ~8 MBq of ^177^Lu-m11B6. Bi-exponential functions were fitted to the data points by a least-square algorithm, and the number of disintegrations (cumulated activity) was calculated as the integral of these expressions multiplied with the decay factor. The cumulated activity *Ã* was thus calculated from

$$ \tilde{A}\kern0.5em =\kern0.5em {\int}_0^{\infty}\kern0.5em A(t)\kern0.5em dt\kern0.5em =\kern0.5em {\int}_0^{\infty}\kern0.5em \left({A}_1{e}^{-{\lambda}_1t}\kern0.5em +\kern0.5em {A}_2{e}^{-{\lambda}_2t}\right){e}^{-{\lambda}_{\mathrm{phys}}t}\kern0.5em dt $$

The activity concentration in the red marrow was supposed to be proportional to that of the blood at a ratio of 0.36 [21]. For determination of S-factors relevant for the animal model, a version of the MOBY phantom [22] was used in which the body weight and the organ sizes could be specified. The average weight, acquired from the kinetic study of each excised organ, was used together with the average total body weight, in order to set each individual organ size in the dosimetry model. A subcutaneous tumor was added on the right flank of the digital phantom (a spherical ellipsoid outside the normal skin contour, with the short axis half to the two long axes and with the short axis perpendicular to the skin). The submandibular gland was manually added to the phantom and represented as a sphere with the radius correlated to the average weight of the organ. The mouse phantom was voxalised in 160 × 160 × 440 voxels and then acted as an input for Monte Carlo simulations of S-factors for ^177^Lu with the MCNPX 2.6 code [23]. The MIRD scheme [24] was applied together with the mouse-specific S-factors for calculation of the organ mean absorbed doses:

$$ {D}_{rT}\kern0.5em =\kern0.5em \underset{rS}{\varSigma}\kern0.5em {D}_{rT\leftarrow rSrT}\kern0.5em =\kern0.5em \underset{rS}{\varSigma}\kern0.5em {\displaystyle {\int}_0^{\infty }A\left(rS,t\right)}\kern0.5em \cdot \kern0.5em S\kern0.5em \left(rT\kern0.5em \leftarrow \kern0.5em rS\right)dt $$

#### Therapy planning

Based on relationships previously determined by Larsson et al. between bone marrow-absorbed dose and biological effects on blood cell counts in rats undergoing ^177^Lu RIT [25], a tolerable absorbed dose for the bone marrow in mice was estimated to be in the order of 12 Gy. This was used in combination with the calculated bone marrow dosimetry to estimate the activity to be administrated.

### Therapy studies

Therapy studies with ^177^Lu-m11B6 were performed on mice with LNCaP xenografts and DU 145 xenografts. Groups of animals with LNCaP xenografts were administered with ^177^Lu-m11B6 aiming at 10 MBq (*n* = 5), 20 MBq (*n* = 5), and 40 MBq (*n* = 3). The control groups were administered with a 20 MBq ^177^Lu-labeled non-specific IgG (*n* = 5), NaCl (*n* = 5), or 30 μg unlabeled 11B6 (*n* = 4). A control group carrying hK2-negative DU 145 xenografts were injected with 20 MBq of ^177^Lu-m11B6 (*n* = 5) and NaCl (*n* = 5). To study the toxicity following treatment, the animals were monitored for weight loss and/or any decline in their general condition. Weight and tumor size were monitored continuously up to 120 days post-injection. Tumor volumes were measured with a caliper [26]. The length (*l*) and width (*w*) of the tumors were measured and the volume (*V*) was calculated as the volume for a rotated ellipsoid [27] as follows:

$$ V\kern0.5em =\kern0.5em \frac{1}{2}\kern0.5em {w}^2l $$

Weight loss of 20 % or a tumor diameter exceeding 15 mm was set as an endpoint.

## Results

### Radiolabelling of CHX-A''-DTPA-m11B6

The radiolabelling yield was 90 % after 2 h, and the radiochemical purity was 99.9 ± 0.15 % after NAP-5 column purification (*n* = 3). The stability of the radioimmunoconjugate was tested in an EDTA surplus of 500 times and was 99 ± 2.2 % (*n* = 3) after 2 weeks of incubation; for PBS, the corresponding value was 97 ± 2.2 % (*n* = 3). The stability in mouse serum was 99 ± 0.76 % (*n* = 3) after 1 week of incubation.

### SPECT imaging

**Fig. 1:**
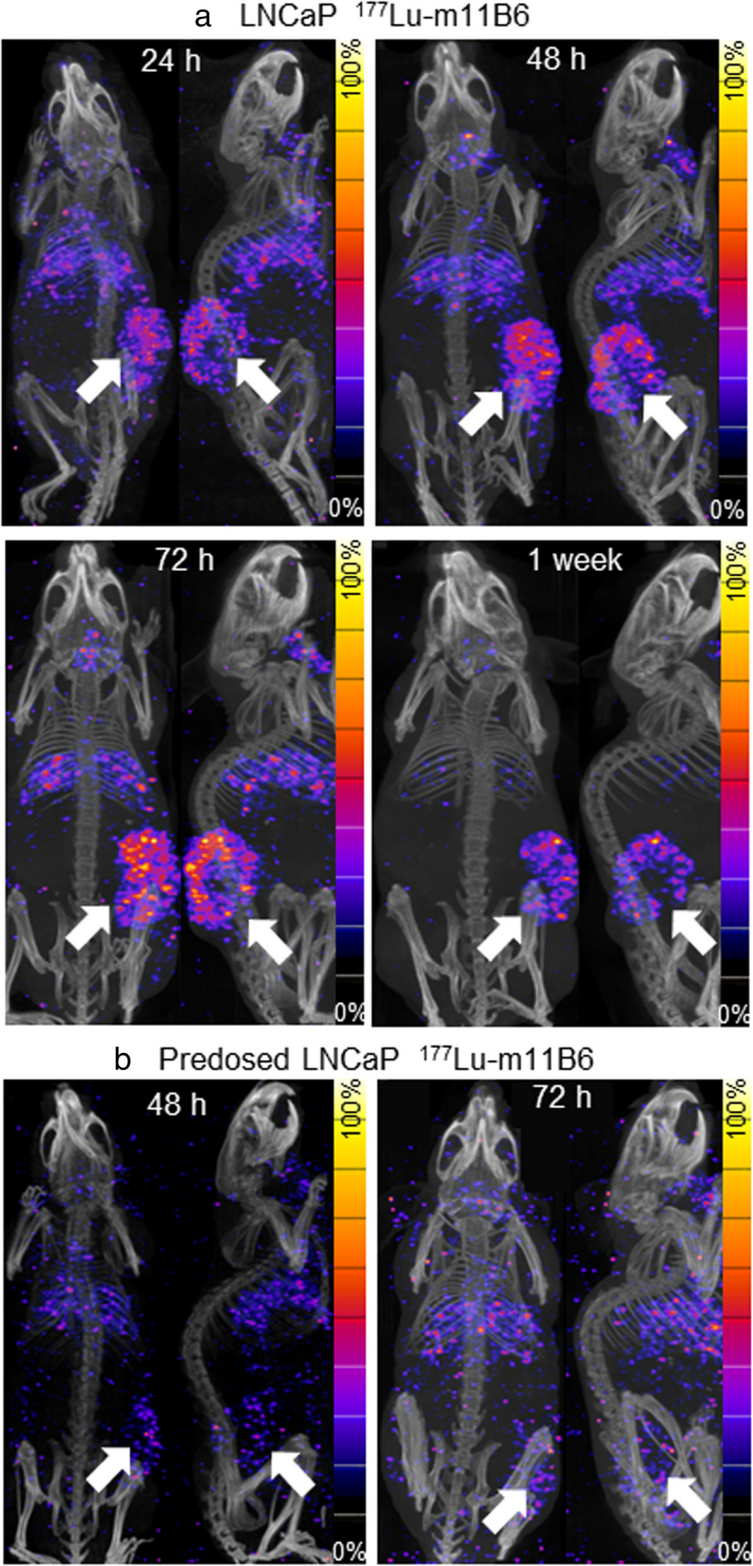
SPECT/CT imaging with two projections per mouse, frontal and sagittal view, *arrows* indicating xenografts. **a** From *left* to *right*, a mouse with LNCaP xenograft (*right side*) imaged at 24, 48, 72, and 1 week p.i. of 8 MBq 177Lu-m11B6. **b** Mouse with LNCaP xenograft (*right side*) imaged at 48 and 72 h p.i. of 8 MBq 177Lu-m11B6 and m11B6. 1 mg cold m11B6 was injected 24 h prior to injection with ^177^Lu-m11B6

**Fig. 2:**
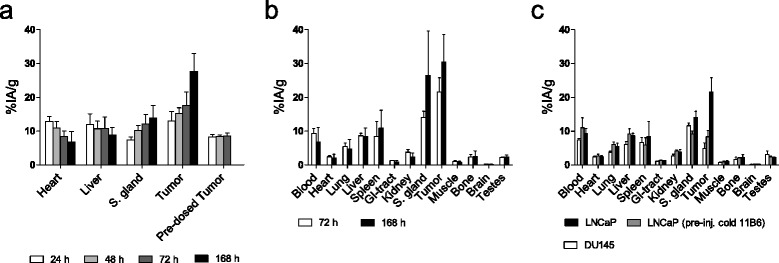
SPECT quantification and biodistribution of 177Lu-m11B6. **a** SPECT quantification of 7.9 ± 0.69 MBq, 30 μg 177Lu-m11B6 in s.c. LNCaP-xenografted NMRI nude mice at 24, 48, 72, and 168 h. **b**. Biodistribution of 7.9 ± 0.69 MBq, 30 μg 177Lu-m11B6 in s.c. LNCaP at 72 and 168 h p.i. **c** In vivo specificity, 7.9 ± 0.69 MBq q, 30 μg 177Lu-m11B6 in s.c. LNCaP- and DU 145-xenografted NMRI nude mice at 72 h with a group of pre-dosed mice (1 mg cold m11B6 24 h pre-injection of labeled antibody)

Activity was accumulated in LNCaP xenografts of nude mice given ^177^Lu-m11B6 up to 1 week post-injection (Fig. 1a). The best contrast was achieved at 1 week post-injection. Tumor uptake was markedly decreased in LNCaP xenografts by pre-injection of cold m11B6 (Fig. 1b). 3D ROIs drawn over the tumor, heart, liver, and submandibular glands gave calculated uptake values of 28 ± 5.3, 6.7 ± 3.1, 8.9 ± 2.2, and 14 ± 3.6 %IA/g, respectively, at 1 week p.i. (*n* = 3) (Fig. 2a). In pre-dosed mice, 3D ROIs over the tumor revealed low or no accumulation with a constant specific uptake at around 8.5 %IA/g at 24, 48, and 72 h. At 72 h, pre-dosed xenografts had 8.6 ± 0.90 compared to 17 ± 4.0 %IA/g for non-blocked (*P* = 0.019) (Fig. 2a).

### Biodistribution

The activity distribution from ex vivo measurements of ^177^Lu-m11B6 is shown in Fig. 2b. Mice injected with ~8 MBq of ^177^Lu-m11B6 showed a tumor accumulation of 22 ± 4.2 %IA/g at 72 h (*n* = 3) and 30 ± 8.2 %IA/g at 168 h (*n* = 3) (Fig. 2b). Distribution of ^177^Lu-m11B6 in LNCaP, DU 145, and pre-dosed LNCaP xenografts showed that uptake was significantly higher in LNCaP than in the control groups, with *P* = 0.003 for DU 145 (4.9 ± 1.6 %IA/g at 72 h) and with *P* = 0.008 for pre-dosed LNCaP xenografts (8.3 ± 1.9 %IA/g, 72 h) (Fig. 2c). This indicates that there is a specific uptake of our labeled radioimmunoconjugate in the non-pre-dosed LNCaP xenografts. There is also a high uptake in the submandibular glands that is not significantly reduced by pre-dosing (Fig. 2c).

### Dosimetry

In Table 1, the calculated absorbed dose per activity unit (Gy/MBq) for ^177^Lu is displayed based on both the biokinetics of ^111^In-m11B6 and of ^177^Lu-m11B6. It was first assumed that an administrated activity of 20 MBq of ^177^Lu-m11B6 would approximately correspond to the absorbed dose of 12 Gy to the bone marrow in mice carrying LNCaP xenografts. This gives an absorbed dose to the tumor of 98 Gy. However, the dosimetric calculations, based on both ^111^In- and ^177^Lu-m11B6 biokinetics, showed that an administrated activity of approximately 27 MBq, would correspond to 12 Gy to the bone marrow and give an absorbed dose to the tumor of 132 Gy, based on ^177^Lu-m11B6 biokinetics. This shows that the use of pre-therapy planning calculating the absorbed dose for determining the activity to be administered can be useful. However, the assumption that ^111^In-m11B6 and ^177^Lu-m11B6 exhibit similar biokinetics appears justified only at the early time points, and at 1 week post-injection, ^177^Lu-m11B6 displays a different curve shape for LNCaP xenograft uptake with a later and higher maximum value [18] resulting in a doubling in absorbed dose per unit activity (Gy/MBq) to the tumor. Estimated absorbed doses for the tumor and some normal organs, where the submandibular glands have the highest calculated absorbed doses, for the administered activities are given in Table 2. It is interesting that there were no observable adverse effects in the group, administrated with 36 MBq of ^177^Lu-m11B6, considering a theoretical absorbed dose in the order of 16 Gy to the bone marrow.

**Table 1:** Organ absorbed dose per injected activity (Gy/MBq) for 177Lu-m11B6 based on A. ^111^In-m11B6 and B. ^177^Lu-m11B6 biokinetics

	A. Based on ^111^In-m11B6		B. Based on ^177^Lu-m11B6	
Organ	Self-dose	Total dose	Self-dose	Total dose
Tumor	2.2	2.3	4.8	4.9
Submand. gland	1.9	2.0	5.6	5.6
Blood	1.3	1.3	1.2	1.3
Bone marrow	0.40	0.47	0.38	0.45
Liver	0.63	0.66	1.56	1.59
Heart	0.47	0.66	0.56	0.74
Lung	0.41	0.54	0.59	0.71
Spleen	0.67	0.71	1.9	1.9
Kidney	0.50	0.53	0.50	0.54
GI tract	0.30	0.35	0.35	0.44
Bone	0.13	0.27	0.18	0.30
Brain	0.017	0.040	0.019	0.041
Testes	0.24	0.28	0.31	0.35

**Table 2:** Estimated absorbed dose (Gy) to tumor and selected normal organs

Organ	10 MBq	19 MBq	36 MBq
Tumor	49	92	180
Submand. gland	56	110	200
Spleen	19	37	70
Liver	16	30	57
Kidney	5.4	10	19
Red bone marrow	4.5	8.6	16

### Radioimmunotherapy

**Fig. 3:**
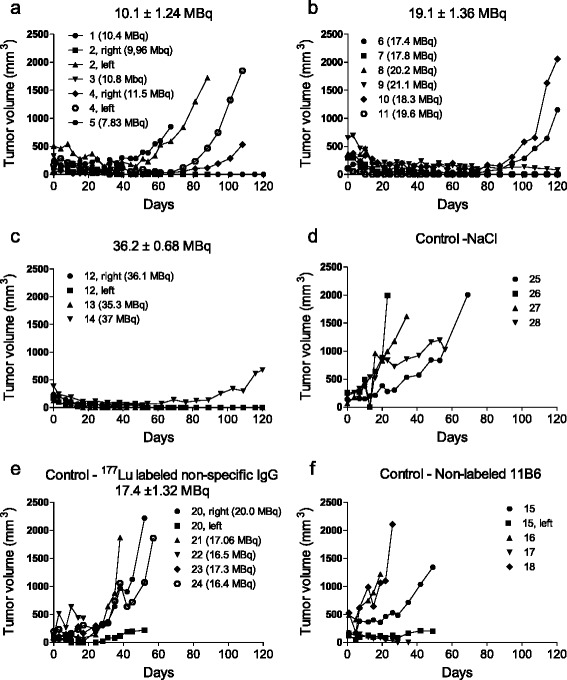
Representation of LNCaP tumor volumes measured with caliper over 120 days after injection of **a** 10.1.2 MBq of 177Lu-m11B6, **b** 19.1.4 MBq 177Lu-m11B6, **c** 36 ± 0.68 MBq 177Lu-m11B6, **d** NaCl, **e** non-specific IgG 18 ± 1.3 MBq and **f** 30 μg m11B6

The injected activities were 10.1 ± 1.4, 19 ± 1.4, and 36 ± 0.7 MBq for the ^177^Lu-m11B6 treatment groups and 17 ± 1.3 MBq for the labeled non-specific IgG control group. There was a clearly visible therapeutic effect in all of the ^177^Lu-m11B6 groups with an onset of tumor shrinkage already present at the first week after injection (Fig. 3a–c), which continued for 2 to 4 weeks after administration.

**Fig. 4:**
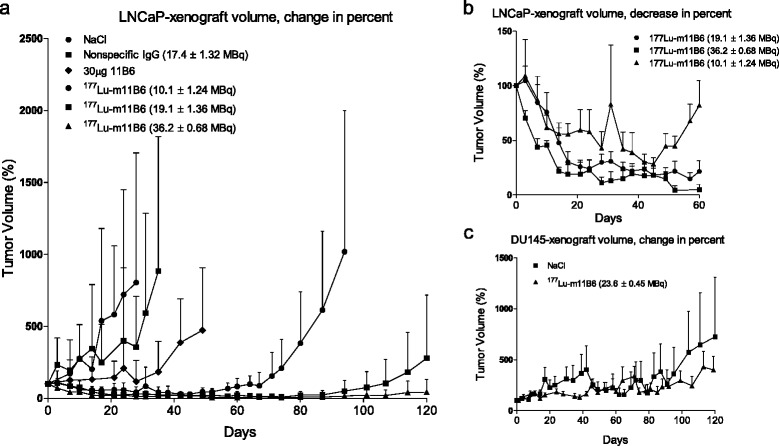
Percent change in xenograft volume over time. **a** Percent change over time 177Lu-m11B6 and controls. **b**. Zoomed in, the change during the first 60 days of 177Lu-m11B6 treatment. **c** Tumor growth development in percent for DU 145 xenografts for mice given 24 ± 0.45 MBq 177Lu-m11B6 and NaCl, respectively

In all control groups, there was an increase of tumor volume after the injection of NaCl, non-labeled m11B6, and ^177^Lu labeled non-specific IgG (Fig. 3d–f). After the initial tumor regression seen in the treated mice, some xenografts became indolent with no tumor growth or shrinkage. Alternatively, a complete remission was observed. Relapses, with recurrent tumor growth, occurred in 40 % of the 19 MBq ^177^Lu-m11B6 group between 40 and 100 days. No relapse was seen in the group administrated with 36 MBq. The therapeutic efficacy of ^177^Lu-m11B6 was dose-dependent, and none or very little therapeutic effect was seen in the control groups as demonstrated by the average tumor growth/shrinkage in percentage of initial tumor volume over time (Fig. 4a, b).

**Fig. 5:**
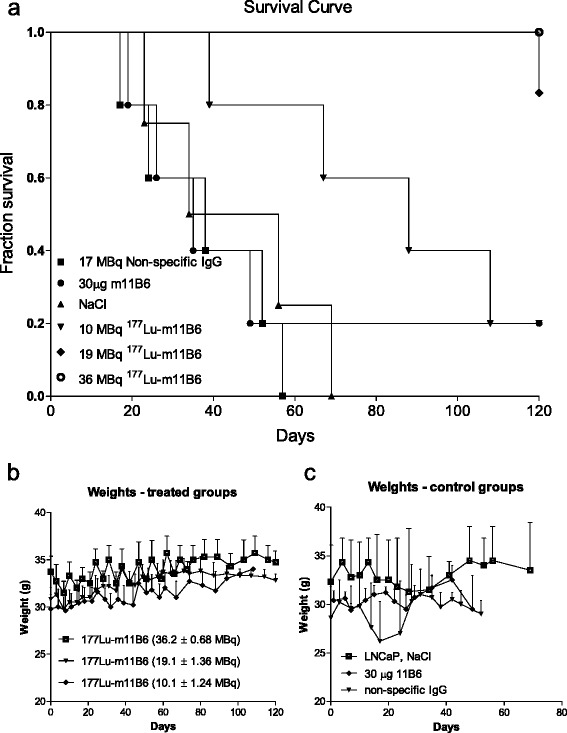
Survival and weight. **a** Kaplan-Meier. Survival over time, median survival was 45 days for NaCl, 38 days for non-specific IgG, 35 days for 30 μg m11B6, and 88 days for 10 MBq of ^177^Lu-m11B6. For the two remaining groups there is no median survival available since only one mouse where sacrificed within 120 days due to too large tumor volume. **b** Animal weight after treatment with ^177^Lu-m11B6, no change is seen. **c** Animal weight in control groups, a drop in the weights of some animals were seen, especially in the group given non-specific IgG

The lowest therapeutic efficacy was observed in the group administrated with 10 ± 1.2 MBq of ^177^Lu-m11B6, but survival was nonetheless prolonged in this group with a median survival of 88 days versus 39 (±5) days for the three control groups (Fig. 5a). For the two other groups treated with higher activity of ^177^Lu-m11B6, it was not possible to calculate a median survival since only one mouse reached the endpoint criteria within 120 days. Interestingly, little toxicity was seen in the ^177^Lu-m11B6 treated groups and weight loss was only seen in the control groups (Fig. 5b), most likely as a result of growing tumor burden and, in the case of the mice treated with non-specific IgG, probably due to high absorbed doses to normal organs (Fig. 5c). Additionally, little therapeutic effects were observed in the control groups carrying hK2-negative DU 145 xenografts that were treated with ^177^Lu-m11B6 (24 ± 0.5 MBq) (Fig. 4c).

## Discussion

In this study, we have demonstrated the therapeutic efficacy of ^177^Lu-m11B6, a hK2-targeting radioimmunoconjugate, in prostate cancer xenografts. ^177^Lu-m11B6 displays interesting therapeutic properties and high uptake in subcutaneous LNCaP xenografts. For the lowest administrated activity of ^177^Lu-m11B6 of 10 MBq, a median survival of 88 days was achieved compared to 39 days median survival on average for the control groups (Fig. 5a). For administrated activities of ^177^Lu-m11B6 in the range of 19 and 36 MBq, the survival was 100 % up to 120 days; a high therapeutic effect attributed to the high estimated absorbed doses delivered to the tumor of 48, 92, and 180 Gy (Table 2). This is in the range of what creates clinical response in a solid tumor, like PCa, using EBRT [28]. In the majority of RIT studies conducted in solid tumors, absorbed doses below 50 Gy are reported [29]; hence, our data for ^177^Lu-m11B6 is promising. Note however that these comparisons should be interpreted with care, due to the difference in modality and subsequent difference in the dose rates given. Still, the lower absorbed dose, 48 Gy, was enough to allow for efficient treatment and with less concern for bone marrow toxicity (4.5 Gy versus 8.6 Gy and 16 Gy, see Table 2).

A potential threat to the use of m11B6 in RIT is that the organ which received the highest absorbed dose was the submandibular glands; however, the uptake was not significantly reduced by pre-dosing (Fig. 2c) which means that it might not be specific. One explanation could be that the high uptake in the submandibular glands of the murine model could be due to cross-reactivity. Murine submandibular glands are known to be abundant with mice kallikreins; however, the KLK2 gene is a silenced pseudogene in mice [30]. In humans, salivary gland tissue immunostaining towards hK2, though positive, has been shown to be considerably less intense than that of the prostate and prostate carcinoma [31].

The maximum tumor uptake of ^177^Lu-m11B6 was higher than previously seen with ^111^In-m11B6, probably due to the use of a higher antibody dose (30 versus 20 μg) [17]. A higher xenograft uptake could in theory contribute to less toxicity. However, the calculated absorbed dose to the bone marrow is similar for both cases. Additionally, several normal organs have theoretically higher absorbed doses with ^177^Lu-m11B6-based dosimetry calculations compared to that with ^111^In-m11B6 (Table 1). Still, even at 16 Gy to the bone marrow, a 4-Gy higher absorbed dose than the predicted tolerable dose, no adverse effects were observed. These findings combined highlight not only the difficulties of predicting toxicity but also the potential of pre-therapy planning, with, e.g., ^111^In labeled immunoconjugates, for finding reasonable ranges of therapeutic activities to be administered in pre-clinical studies. Pre-therapy dose planning, as done in clinical studies, could maybe replace the maximum tolerated dose or the maximum tolerated activity studies in pre-clinical therapy studies.

RIT in general has so far not been very successful in solid tumors due to the limited penetration of antibodies. RIT with PSMA targeting has however shown promise in phase II clinical trials [7]. This PSMA is not only confined to the cells of the prostate but is also expressed in other organs such as the duodenum, the kidneys, the brain, and, like hK2, the salivary glands [32]. Small molecules, labeled with therapeutic radionuclides, targeting PSMA have been developed, e.g., in a recent study, it shown to be effective with limited toxicity to normal organs, including the salivary glands [33]. In the specific case of hK2 targeting, it is not clear how the size of the antibody and the presence of a Fc region affects the uptake and biokinetics of the radioimmunoconjugate. This would have to be further investigated before making any decisions on whether to proceed with testing smaller proteins or other small molecules. Even though smaller molecules could increase the tumor penetration and lower the absorbed dose to normal organs due to faster clearance, the longer retention time of a full antibody contributes to a higher tumor uptake. Schmidt et al. used theoretical analysis to predict tumor uptake in relation to size and affinity [34]. Their model showed that tracers with the smallest and largest molecular masses exhibited the highest tumor uptake, whereas tracers with intermediate mass (25–60 kDa) displayed the lowest tumor uptake. It has been shown that high-affinity radioimmunoconjugates (low *K*_D_ values) generally have low penetration and reduced diffusion due to the so-called binding site barrier [35], known to limit therapeutic efficacy. An affinity of 1 nM has been considered to be optimal in this type of therapeutic setting [36], but m11B6, being a high-affinity antibody (*K*_D_ of 0.65 pM), has here shown high tumor uptake and good therapeutic efficacy. For the specific case of shedded antigens and high-affinity binders, Pak et al. has suggested that this combination might circumvent the binding site barrier issues seen with high-affinity binders [37]. The fact that hK2 is a secreted antigen [38] could therefore potentially increase its availability for targeting and, in combination with the high affinity of ^177^Lu-m11B6, lead to better tumor penetration.

hK2 can be found in the serum of patients, and the serum levels of hK2 have been found to vary with degree of disease, e.g., Stephan et al. found that grade G3 cancers with a Gleason score larger than 7 had a hK2 median of 0.23 μg/L [39]. Another study by Steuber et al. reported values roughly half of that for patients with extracapsular extension or seminal vesicle invasion [40]. It is not clear how serum levels, albeit low, of hK2 would affect RIT towards the antigen. However, these levels can be continuously monitored.

Due to the problem with resistance, against chemotherapy and targeted therapies, and the complex biology behind advanced PCa, the only reasonable approach is most likely a multi-combinatorial therapy rationale, an approach that comes with the necessity of expanding the clinical therapy options. As a novel therapy target, hK2 has several possible advantages: it is highly restricted, although not completely, to prostatic tissue and it has a possibly higher expression in some patients following EBRT [41] and a seemingly retained expression in the different clinical phases of PCa. It is therefore plausible to assume that the use of hK2 targeting therapy might not be limited to only one stage of the disease. And, even though it has not yet been investigated in a model of castration-resistant disease, it could potentially be useful in CPRC patients as well as in patients undergoing treatment for disseminated androgen responsive disease.

More studies are needed in order to further investigate the underlying factors and mechanisms behind the therapeutic effects of ^177^Lu-m11B6, but the current results present a radioimmunoconjugate with high potential for the treatment of prostate cancer. Remarkably, an administrated activity of 10 MBq showed a survival of 88 days, and a twofold increase of the administered activity gave a 100 % survival in this group up to 120 days. For future intended use in humans, we are now working on a new less immunogenic humanized version of the radioimmunoconjugate.

## Conclusions

Here, we demonstrate the encouraging therapeutic efficacy of ^177^Lu-m11B6, targeting the human kallikrein-related peptidase 2, with low observed adverse effects. We also investigated the concept of pre-therapy dosimetry planning in pre-clinical studies with interesting results regarding the predicted tolerable absorbed dose to the bone marrow.
